# Ultra-High Dose Rate Electron Beam Dosimetry Using Ag Nanoparticle-Enhanced nPAG and NIBMAGAT Gels

**DOI:** 10.3390/gels11050336

**Published:** 2025-04-30

**Authors:** Mantvydas Merkis, Akvile Slektaite-Kisone, Marius Burkanas, Aleksandras Cicinas, Mindaugas Dziugelis, Vaidas Klimkevicius, Diana Adliene, Jonas Venius

**Affiliations:** 1 Department of Physics, Kaunas University of Technology, Studentų g. 50, LT-51368 Kaunas, Lithuania; 2 Medical Physics Department, National Cancer Center, Affiliate of Vilnius University Hospital Santaros Klinikos, Santariškių g. 1, LT-08406 Vilnius, Lithuania; 3 Institute of Chemistry, Vilnius University, Naugarduko g. 24, LT-03225 Vilnius, Lithuania; 4 Biomedical Physics Laboratory, National Cancer Institute, P. Baublio g. 3b, LT-08406 Vilnius, Lithuania

**Keywords:** FLASH, gel dosimetry, radiotherapy

## Abstract

FLASH radiation therapy is an emerging technique that provides several advantages over conventional radiotherapy. By delivering ultra-high dose rate radiation, the damage to healthy tissues surrounding the treatment area is minimized, treatment time is reduced and treatment outcomes of radioresistant tumors are improved. Despite its promising potential, FLASH radiation therapy remains relatively understudied, particularly in the field of dosimetry. Polymer gel dosimetry is a promising technique for verifying FLASH radiation therapy because it enables volumetric dose distribution measurements with high spatial accuracy. This study investigates the applicability of two commonly used polymer gel dosimeters, nPAG and NIBMAGAT, enhanced with nanoparticles, in ultra-high dose rate radiation therapy. The results indicate that NIBMAGAT gel, enriched with Ag nanoparticles, outperforms nPAG. NIBMAGAT gel exhibits less saturation at high doses, maintains dose rate independence and offers comparable sensitivity to nPAG formulation.

## 1. Introduction

FLASH radiation therapy (FLASH-RT) is an advanced radiotherapy technique that delivers ultra-high dose rate (UHDR) radiation, typically exceeding 40 Gy/s. Compared to conventional radiation therapy (CONV-RT), this method minimizes damage to surrounding healthy tissues while retaining efficient tumor control [1]. Its potential has been validated in animal studies including rodents, cats and canines across various radiosensitive organs such as the lungs, intestine, brain and others [2]. In 2019, the first human patient with multiresistant T-cell lymphoma was successfully treated with FLASH-RT, showing positive outcomes [3]. Currently, several clinical trials using electron and proton-based FLASH-RT are underway, exploring its broader applicability in cancer care [4].

The dose rate is a critical aspect of FLASH-RT in achieving its unique therapeutic benefits. The application of UHDR radiation can result in a dose-modifying factor ranging from 1.2 to 1.6, depending on the absolute dose, dose rate, tissue type, radiation beam characteristics, etc. [5]. Theoretically, FLASH-RT allows for the delivery of higher doses than CONV-RT without increasing toxicity, but requires high precision to ensure that both the administered dose and the dose rate remain within optimal ranges.

Despite the substantial advantages of FLASH-RT, high dose rate dosimetry is poorly investigated. Taking into account that polymer dosimetric gels are almost tissue equivalent and can provide 3D dose distribution in the target with very high spatial resolution, mainly depending on the readout method, gel dosimeters could be particularly valuable for radiotherapy treatment plan verification [6]. Some preliminary results were obtained using NIPAM dosimetric gel, which showed signs of dose response saturation and severe dose rate dependence when analyzing FLASH-RT doses, indicating the need for further investigations [7].

The working principle of polymer gel dosimeters relies on radiation-induced polymerization reactions, directly depending on the absorbed dose [8]. Upon exposure to ionizing radiation, the water within the dosimetric gel undergoes dissociation, generating reactive radicals. Generated radicals interact with monomers or polymers, forming monomer/polymer radicals. Subsequently, these radicals react with other polymers and monomers, leading to the formation of polymer chains or networks. Radiation-induced polymerization results in alterations to the physical properties of the dosimetric gels, such as changes in optical density or physical density [9]. Dose information, registered by polymer gel dosimeters, can be read using various techniques, such as magnetic resonance imaging (MRI), computed tomography (CT) or optical measurements. Currently, MRI is considered the gold standard technique for polymer gel analysis due to its high sensitivity [6].

Typical dosimetric gel consists of monomers, crosslinkers, scaffolding, oxygen scavengers and water [10]. Monomers and crosslinkers are precursors for radiation-induced polymerization reactions. The structure of the gel is maintained using a scaffolding agent, typically gelatin. An oxygen scavenger reduces the inhibitory effect of oxygen in polymerization reactions. Water serves as the main solvent and source of water radicals produced during the radiolysis process [9]. The main differences between various polymer gel formulations lie in the type of monomers used. The most common monomers are acrylamide and methacrylic acid [9], which are highly toxic (especially acrylamide). Therefore, alternative monomers are being investigated, such as *N*-isopropylacrylamide or *N*-(isobutoxymethyl)acrylamide [11,12].

Dose response sensitivity of polymer gel dosimeters could be improved using various additives, for example: iodine [13], urea [14], co-solvents [9,15], glucose [16] and inorganic salts [17]. Using nanoparticles to enhance the sensitivity of polymer gel dosimeters is one of the most promising options. Dose sensitivity enhancement in the presence of metal nanoparticles in polymer gels is possible because the probability of radiation interaction is higher due to the high surface to volume ratio of nanoparticles which leads to an intensifying photoelectric absorption effect [18,19]. Gold nanoparticles are most commonly used for this purpose. Farahani et al. modified methacrylic acid based MAGAT dosimetric gel with gold nanoparticles and achieved a dose sensitivity enhancement of 15.3% when irradiation was performed with an Ir-192 (380 keV) source [20]. Mahdavi et al. achieved dose sensitivity enhancement of 10% when gold nanoparticle additive was used with MAGICA dosimetric gel under 18 MeV irradiation [21]. However, other nanoparticles could also be used as a viable option. Sabbaghizadeh et al. achieved an 11.82% dose response increase adding Ag nanoparticle to a PAGAT dosimetric gel irradiated with 1.25 MeV photons [22].

In this work, we investigated the response of two commonly used dosimetric gels, nPAG and NIBMAGAT, under UHDR irradiation. The nPAG formulation is well-established in various publications [23,24,25,26]. However, a significant drawback of this gel is the use of the toxic monomer acrylamide. Because of this, an alternative dosimetric gel formulation was investigated. It was found that one of the most promising low-toxicity alternatives is NIBMAGAT gel, where acrylamide is replaced with the less toxic *N*-(isobutoxymethyl)acrylamide. Additionally, the response of dosimetric gels enriched with nanoparticles was investigated under UHDR irradiation, as previous research has demonstrated significant improvements in the response under conventional dose rate (CONV) irradiation [9,27].

## 2. Results and Discussion

### 2.1. Dose Response Analysis

**Figure 1:**
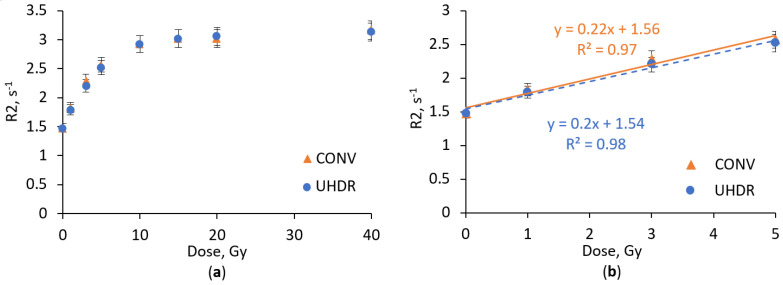
Dose response of nPAG dosimetric gel irradiated with UHDR and CONV electron beam in the dose range up to 40 Gy (**a**) and up to 5 Gy (**b**).

The response of the nPAG dosimetric gel under CONV and UHDR electron irradiation is shown in Figure 1. The response of the nPAG dosimetric gel remained similar for both irradiation types, demonstrating dose response stability under high variations in irradiation dose rate. The acquired results are comparable with findings of other authors who investigated dose rate effect, although other studies utilized much lower irradiation dose rates. Sellakumar et al. [28] did not report any response differences in a similar PAGAT dosimetric gel under photon irradiation with dose rates varying from 0.5 to 5 Gy/min. Similar results were demonstrated by Zehtabian et al., who demonstrated that the response of the PAGAT dosimetric gel was stable in the low irradiation dose rate range of 2–8 Gy/h [29].

Noticeable saturation effects at doses exceeding 5 Gy were observed (Figure 1a). These effects are similar to those reported in other studies on acrylamide-based polymer gels [28,30]. However, in the dose range up to 5 Gy, the nPAG dosimetric gel response could be approximated with linear dependency. In this range, the dosimetric gel exhibited similar sensitivities of 0.22 s^−1^Gy^−1^ and 0.2 s^−1^Gy^−1^ to CONV and UHDR irradiation, respectively (Figure 1b). These results are comparable with the findings in other investigations [23].

**Figure 2:**
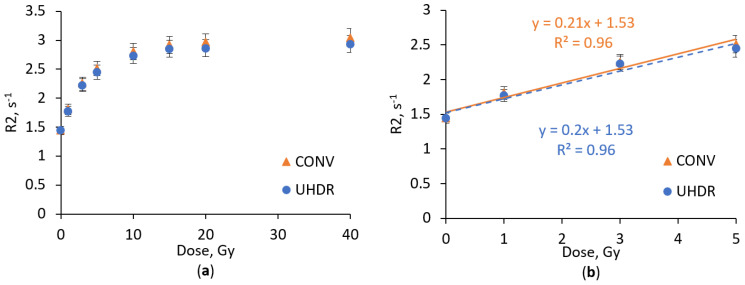
Dose response of nPAG + Ag dosimetric gel irradiated with UHDR and CONV electron beam in the dose range up to 40 Gy (**a**) and up to 5 Gy (**b**).

Since metal nanoparticles have been used in several studies [9,27] to increase the dose response sensitivity of polymer gel dosimeters, we decided to use Ag nanoparticles to increase the sensitivity of the nPAG dosimetric gel (Figure 2).

Compared to the conventional nPAG dosimetric gel formulation, the nPAG + Ag gel indicated similar saturation tendency at doses >5 Gy for both (CONV or UHDR) irradiation techniques (Figure 2a). In the dose range up to 5 Gy (Figure 2b) it was noted that the addition of Ag nanoparticles did not significantly influence the sensitivity of the dosimetric gel. Sensitivities of gels with Ag NPs additives irradiated with different dose rate radiation reached 0.21 s^−1^Gy^−1^ and 0.2 s^−1^Gy^−1^ for CONV and UHDR techniques, respectively, also demonstrating the low dose rate dependency of the formulation. Since no significant decrease in sensitivity of the dosimetric gel with nanoparticle additives was identified, we concluded that the used concentration of nanoparticles and usage of stabilizer trisodium citrate prevents the aggregation of nanoparticles and associated sensitivity diminishing effect [31]. No sensitivity enhancement effect using Ag nanoparticles may be attributed to the irradiation energy used (6 MeV), where the Compton effect dominates over the photoelectric effect. It is known that only the probability of the photoelectric effect depends on the atomic number of the material (∝ Z^5^), leading to a significant increase in interaction probability when high atomic number additives are used. This theory is supported by the findings of Rahman et al., who observed a reduced sensitivity-enhancing effect of Au nanoparticles in the nPAG dosimetric gel when the irradiation energy was increased [32].

**Figure 3:**
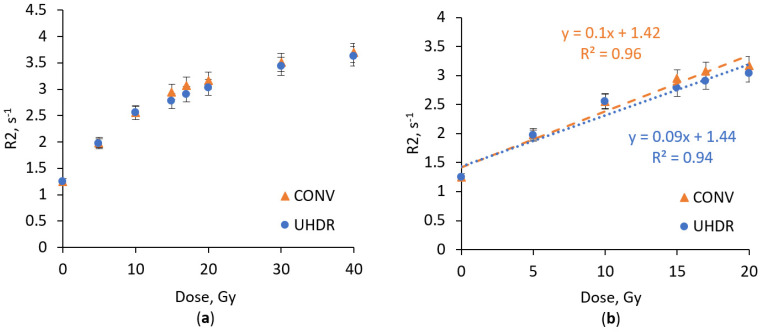
Dose response of NIBMAGAT dosimetric gel irradiated with UHDR and CONV electron beam in the dose range up to 40 Gy (**a**) and up to 20 Gy (**b**).

Since toxic acrylamide is one of the most important components of the nPAG dosimetric gel, application of the less toxic alternative, *N*-(isobutoxymethyl)acrylamide, was suggested to replace acrylamide [33]. The dose response of the NIBMAGAT dosimetric gel containing *N*-(isobutoxymethyl)acrylamide irradiated under CONV and UHDR conditions is shown in Figure 3.

The NIBMAGAT gel offers a significant advantage compared to the well-established nPAG dosimetric gel: the NIBMAGAT dosimeter exhibits less significant saturation effects, regardless of changes in the dose rate. Under both irradiation conditions the response was approximated with linear dependency in the dose range up to 20 Gy. High dose response linearity under CONV irradiation was also demonstrated by Basfar et al. [33].

**Figure 4:**
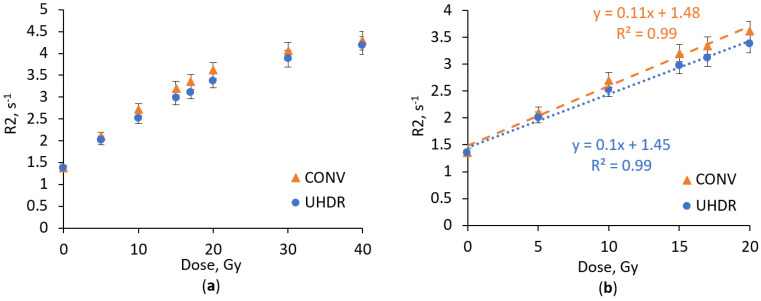
Dose response of NIBMAGAT + Ag dosimetric gel irradiated with UHDR and CONV electron beam in the dose range up to 40 Gy (**a**) and up to 20 Gy (**b**).

In terms of dose sensitivity, the NIBMAGAT dosimetric gel is considerably less sensitive compared to the nPAG formulation. Under CONV and UHDR irradiation, the NIBMAGAT gel exhibited sensitivities of 0.1 s^−1^Gy^−1^ and 0.09 s^−1^Gy^−1^, respectively. In contrast, the nPAG gel reached sensitivities of 0.22 s^−1^Gy^−1^ and 0.2 s^−1^Gy^−1^. The NIBMAGAT dosimetric gel exhibits minimal dose rate dependency, with only slight changes in the sensitivity, offset and linearity when switching from CONV to UHDR irradiation. Rabaeh et al. also confirmed that dose rate variations have a negligible impact on the response of the NIBMAGAT gel [11]. In order to enhance the relatively low sensitivity of the NIBMAGAT gel, Ag nanoparticles were incorporated into the gel. Figure 4 shows dose–response curves of NIBMAGAT + Ag gel under UHDR and CONV irradiation conditions.

Dose–response curves demonstrate that the addition of Ag nanoparticles enhance the dose sensitivity of NIBMAGAT dosimetric gel. The sensitivity of Ag nanoparticle-enriched NIBMAGAT gel increased from 0.1 s^−1^ Gy^−1^ to 0.11 s^−1^Gy^−1^ (15%) under CONV irradiation, and from 0.09 s^−1^Gy^−1^ to 0.1 s^−1^Gy^−1^ (14%) under UHDR irradiation. Notably, sensitivity enhancement remained similar despite the considerable difference in irradiation dose rate. Also, the Ag nanoparticle-enriched NIBMAGAT gel exhibited almost perfectly linear (R^2^ = 0.99) response up to 20 Gy, with low irradiation dose rate dependency.

More effective dose sensitivity enhancement by adding Ag nanoparticles to the NIBMAGAT gel, as compared to the nPAG gel, can be attributed to the improved solubility of the crosslinker due to the presence of acetone as a co-solvent. This enables broader participation of nanoparticles in interaction with water radicals during polymerization process. A similar conclusion was drawn by Lotfy et al., who suggested that increasing the crosslinker concentration can lead to an increase in the dose response of dosimetric gel [34].

Generally, the NIBMAGAT dosimetric gel demonstrates superior characteristics for UHDR dosimetry compared to the nPAG gel despite the fact that the dose sensitivity is lower. The NIBMAGAT gel exhibits a lower saturation tendency of dose sensitivity at higher doses. The incorporation of nanoparticles into the NIBMAGAT gel allows it to achieve the dose sensitivity which is comparable with the sensitivity of more toxic acrylamide-based nPAG gel. In terms of dose rate dependency, all investigated dosimetric gel formulations demonstrated only minor changes in the response despite dramatic changes in the dose rate. Notably, the sensitivity of all investigated dosimetric gel formulations under CONV irradiation was slightly higher compared to UHDR irradiation. This effect could be attributed to possible polymer degradation under UHDR irradiation.

### 2.2. FTIR Analysis

Although the dosimetric properties of nPAG and NIBMAGAT gels for UHDR beam dosimetry have been investigated, there is still a gap in understanding of their molecular structure differences due to irradiation with different dose rates. To address this, ATR-FTIR spectroscopy measurements of dosimetric gel samples irradiated using both CONV and UHDR irradiation techniques have been performed.

**Figure 5:**
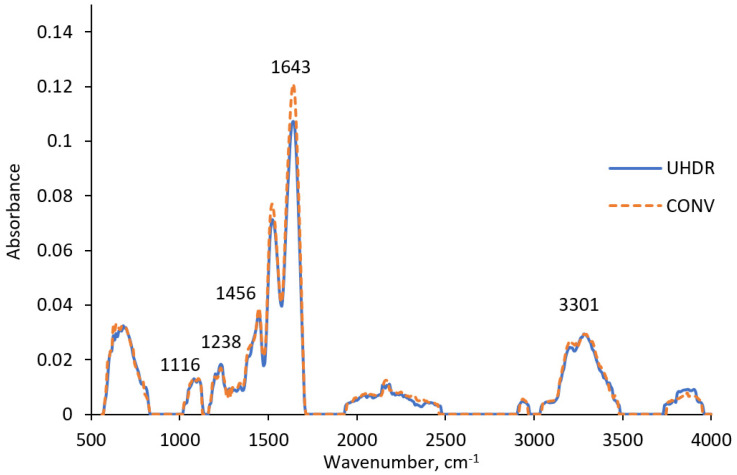
ATR-FTIR spectra of nPAG dosimetric gel irradiated with UHDR and CONV electrons.

A comparison of ATR-FTIR spectra of nPAG dosimetric gel samples irradiated with CONV and UHDR beams (Figure 5) revealed noticeable differences at 1643 cm^−1^, suggesting a more rapid reduction of the C=C bonds at higher dose rates [35]. Taking into account that C=C bonds are converted to C-C bonds during polymerization, it can be concluded that irradiating nPAG gel with higher dose rate may intensify polymerization reactions [36,37]. However, this process has not affected the dose–response of the nPAG gel, which exhibited slightly higher sensitivity under CONV compared to UHDR irradiation (Figure 1b). Other ATR-FTIR peaks did not show significant differences between UHDR and CONV irradiation types. Peaks at 1116 cm^−1^ and 1238 cm^−1^ could be associated with C-N stretching vibrations present in the gelatin, monomer and crosslinker [38]. The peak at 1456 cm^−1^ corresponds to C-H bending vibrations present in most components of the dosimetric gel. O-H stretching vibrations can be observed at 3301 cm^−1^, indicating the presence of water or water radicals [39].

**Figure 6:**
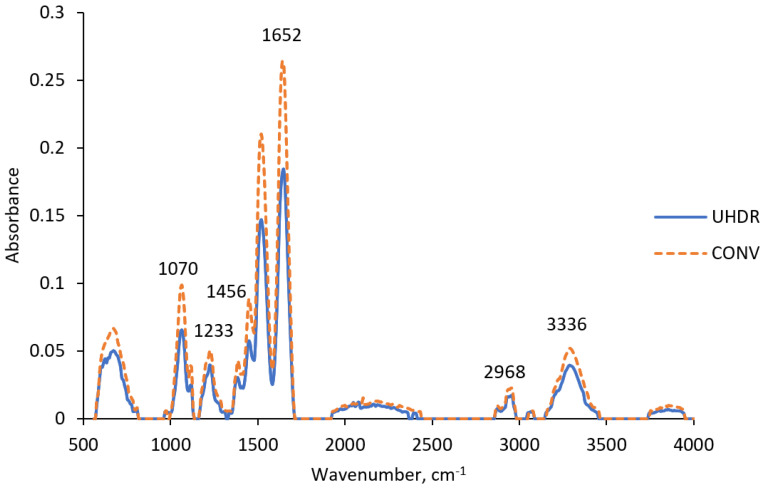
ATR-FTIR spectra of NIBMAGAT dosimetric gel irradiated with UHDR and CONV electrons.

The ATR-FTIR spectrum of the NIBMAGAT dosimetric gel irradiated with UHDR and CONV radiation exhibited more pronounced changes compared to the nPAG gel (Figure 6). The most significant changes were observed at 1652 cm^−1^, where a reduction in the intensity of C=C stretching vibrations was noted when switching from CONV to UHDR irradiation [35]. As in the previous case, this indicates a more intense polymerization process under UHDR irradiation, as more C=C bonds are converted to C-C bonds [36]. This finding was not reflected in the acquired dose–response curves of the dosimetric gel (Figure 3b), suggesting that differences in polymer formation processes, depending on the irradiation dose rate, are relatively insignificant in the NIBMAGAT dosimetric gel.

Peaks at 1070 cm^−1^ and 1233 cm^−1^ correspond to C-N stretching vibrations. However, the increase in peak amplitude under CONV irradiation cannot be definitively attributed to specific molecular changes, as C-N stretching vibrations occur in various components such as gelatin, monomer, or crosslinker [38]. A similar situation occurs with the C-H bending peak at 1456 cm^−1^, which could be associated with the higher dissociation of a double bond in the monomer under CONV irradiation. This finding could be supported by dose–response data, where slightly higher sensitivity was notified under CONV irradiation conditions. However, because C-H bending is present in many other dosimetric gel components, a definitive conclusion cannot be drawn [39]. N-H stretching vibrations were observed at 2968 cm^−1^ and could be associated with gelatin. However, no significant differences were observed between different irradiation types. Slight indications of higher water radical generation under CONV irradiation were noted at 3336 cm^−1^, where more intense O-H stretching vibrations were detected [39].

### 2.3. Uncertainty Budget

The sources of uncertainties in this study were: irradiation, sensitivity variation of imaging method, dose rate dependency, calibration curve fit and reproducibility. Similar sources of uncertainties were also identified by other authors [40,41,42]. Evaluation of uncertainties was performed analyzing acquired data using methodology described by [41,43] and utilizing results of other investigations [44,45].

According to the performed analysis (Table 1), all investigated dosimetric gel formulations demonstrated similar uncertainties, with the highest combined uncertainty of 5.6% for the nPAG + Ag dosimetric gel and the lowest of 5% for the initial nPAG dosimetric gel. Differences between formulations were mainly influenced by the uncertainty of the calibration curve fit and dose rate dependency, which was not substantial—the maximum dose rate related uncertainty was 2.5%.

## 3. Conclusions

An investigation of nanoparticle enriched nPAG and NIBMAGAT dosimetric gels irradiated under CONV and UHDR irradiation conditions has shown that the NIBMAGAT gel enriched with Ag nanoparticles is a promising candidate for UHDR beam dosimetry. The NIBMAGAT + Ag gel exhibited linear dose response tendency in the broad interval of doses up to 20 Gy with small saturation; the response is almost dose-rate independent, and the sensitivity is comparable to the more toxic nPAG gel.

Investigated nPAG and NIBMAGAT dosimetric gels indicated several molecular structure changes related to more intense polymerization processes induced by UHDR irradiation of samples. However, these changes were considered insignificant as they were not definitively reflected in the dose–response curves.

## 4. Materials and Methods

### 4.1. Fabrication of Dosimetric Gel

The nPAG dosimetric gel was prepared according to the procedure described by Venning et al. [46]. Firstly, gelatin (5 wt%, Bloom 300, Sigma-Aldrich, St. Louis, MO, United States) was soaked in distilled water (89 wt%, Eurochemicals, Vilnius, Lithuania) for 10 min and then heated on a magnetic stirrer (Heidolph MR HEI-Standard, Heidolph Instruments, Schwabach, Germany) up to 48 °C. Subsequently, *N,N*′-methylenebis(acrylamide) (3 wt%, Sigma-Aldrich, St. Louis, MO, USA) was added, and the mixture was continuously stirred until fully dissolved. After that, acrylamide (3 wt%, Sigma-Aldrich, St. Louis, MO, USA) was added while maintaining stirring and heating. When the acrylamide dissolved, tetrakis (hydroxymethyl)phosphonium chloride (5 mM, Sigma-Aldrich, St. Louis, MO, USA) was added to the gel and stirred for 2 min. The prepared dosimetric gel was poured into polymethyl methacrylate (PMMA) cuvettes and refrigerated at 6 °C for 24 h to solidify.

The NIBMAGAT dosimetric gel was prepared according to the procedures described by Lotfy et al. [34] and Rabaeh et al. [11]. Firstly, gelatin (4 wt%, Bloom 300, Sigma-Aldrich, St. Louis, MO, United States) was soaked in distilled water (40.8 wt%, Eurochemicals, Vilnius, Lithuania) for 15 min and then heated to 48 °C. When the gelatin fully dissolved, acetone (20 wt%, Eurochemicals, Vilnius, Lithuania) was added to the solution. Once cooled to 40 °C, *N,N*′-methylenebis(acrylamide) (3 wt%, Sigma-Aldrich, St. Louis, MO, USA) was added under continuous stirring and heating for 45 min until full dissolution of the components. After that, *N*-(isobutoxymethyl)acrylamide (2 wt%, Sigma-Aldrich, St. Louis, MO, USA) was added, continuing stirring until dissolution. The prepared solution was cooled down to 35 °C and tetrakis (hydroxymethyl)phosphonium chloride (5 mM, Sigma-Aldrich, St. Louis, MO, USA) was added under continuous stirring for the next 2 min and then the gel was dispensed into PMMA cuvettes. Cuvettes with the dosimetric gel were refrigerated at 6 °C for 24 h to solidify.

Preparation of dosimetric gels with nanoparticle additives was performed following the same procedures described above, but the nanoparticle solution was added to the dosimetric gel before adding the oxygen scavenger (tetrakis (hydroxymethyl)phosphonium chloride). After the addition of nanoparticle solution, the gel was stirred for a couple of minutes. The formulations of the investigated dosimetric gels are summarized in Table 2.

**Figure 7:**
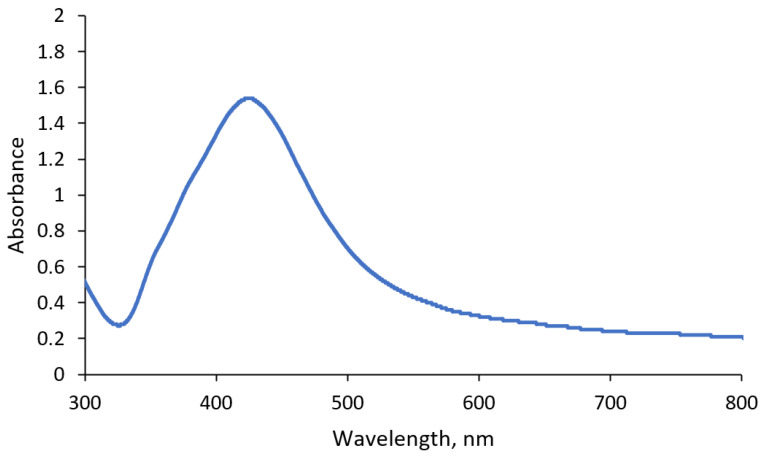
UV–Vis spectrum of synthesized nanoparticle solution.

The nanoparticle solution was prepared from trisodium citrate using the wet chemical reduction method as described by Sileikaite et al. [47]. At first, 45 mg of AgNO_3_ (Eurochemicals, Vilnius, Lithuania) was added to 250 mL of distilled water (Eurochemicals, Vilnius, Lithuania). The solution was stirred and heated using a magnetic stirrer (Heidolph MR HEI-Standard, Heidolph Instruments, Schwabach, Germany) until the boiling point was reached. When the required temperature was reached, 1 mL of 1 wt% trisodium citrate (Sigma-Aldrich, St. Louis, MO, USA) was added to the solution. Stirring and heating continued for 10 min until the color changed to yellow. The presence of nanoparticles was verified using UV–Vis spectroscopy (Ocean Optics USB4000, Orlando, FL, USA). The resulting UV–Vis spectrum showed a large, localized surface plasmon resonance peak at 430 nm, corresponding to nanoparticles with an average size of 60 nm (Figure 7) [48].

### 4.2. Irradiation of Dosimetric Gels

The irradiation of dosimetric gel samples was performed in Varian TrueBeam (Varian Medical Systems, Palo Alto, USA) linear accelerator (LINAC). For irradiation with conventional (CONV) dose rate (3 Gy/min), the following parameters were used: 6 MeV electron beam, 15 × 15 cm^2^ field size and 100 cm source-surface distance. Irradiation doses were 0 Gy, 1 Gy, 3 Gy, 5 Gy, 10 Gy, 15 Gy, 20 Gy and 40 Gy for the nPAG gel and 0 Gy, 5 Gy, 10 Gy, 15 Gy, 17 Gy, 20 Gy, 30 Gy and 40 Gy for the NIBMAGAT gel, taking into account expected differences in dose response.

**Figure 8:**
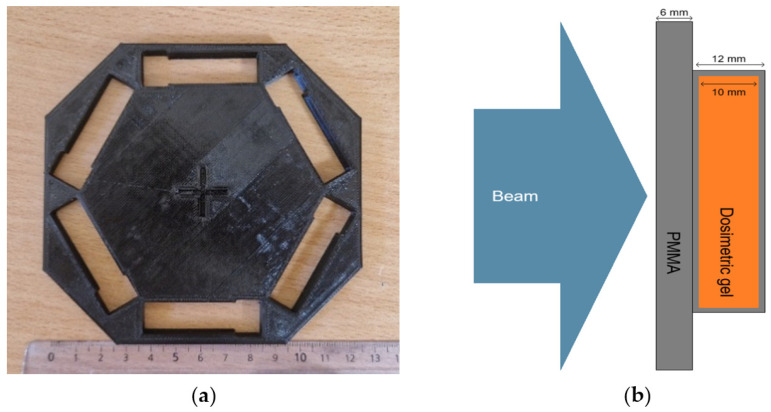
Cuvette adapter, used for the irradiation (**a**); dosimetric sample irradiation scheme (**b**).

For the irradiation of dosimetric gel samples using UHDR electrons, a modified Varian TrueBeam LINAC was employed, as described by Slektaite-Kisone et al. [49]. Specifically, the monitor chamber, flattening filter and target were removed from the beam path in the LINAC. Irradiation was conducted using 6 MeV photon beam settings for the system parameters. The dose control unit consisted of 3 parts—the photodiode, amplifier and microcontroller. The Cherenkov pulses were registered by photodiodes, then amplified and finally counted by a microcontroller. After achieving a certain number of pulses, the microcontroller turned off the beam. UHDR beam field was round-shaped and 20 cm in diameter. Samples were irradiated using a 3D-printed adapter (Figure 8a) according to the scheme provided in Figure 8b. Each pulse delivered 1 Gy dose with a duration of 3.6 µs. The estimated instantaneous dose rate of the generated electron beam was approximately ~0.27 MGy/s.

**Figure 9:**
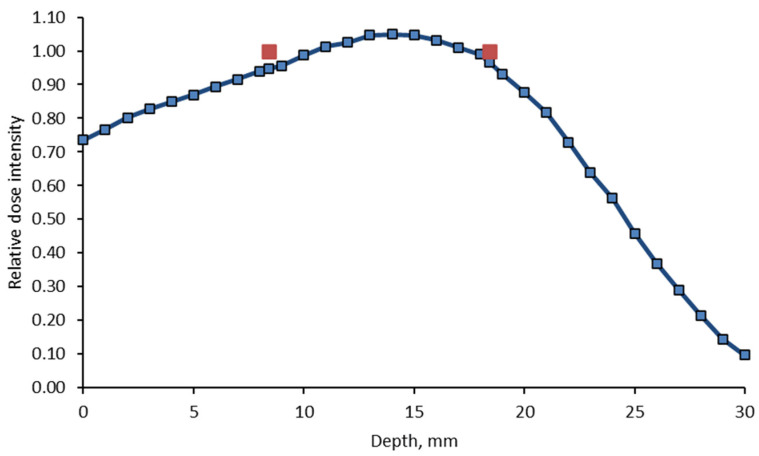
Percent depth dose (PDD) curve in water, 6 MeV electron beam. Red dots indicate the irradiated sample location.

The percent depth dose (PDD) curve was measured for LINAC during commissioning as well as during periodic quality assurance testing. According to the 6 MeV electron PDD curve (Figure 9), the location of the cuvette was selected in a water-equivalent depth where the irradiation is the most uniform. The irradiation position of the samples in the PDD curve was marked with red dots. This was realized using a PMMA sheet of appropriate thickness placed on the dosimetric gel samples (Figure 8b).

UHDR electron irradiation dose verification was performed using EBT4 radiochromic films (Gafchromic, Ashland Advanced Materials, Bridgewater, NJ, USA) [50]. These films were calibrated according to the conventional electron irradiation method. Absolute dose during EBT4 radiochromic film irradiation was measured using the Markus chamber (PTW, Freiburg, Germany). Dosimetric gel samples were irradiated under the same conditions, except for the dose rate.

### 4.3. Analysis of Dosimetric Gel Samples

**Figure 10:**
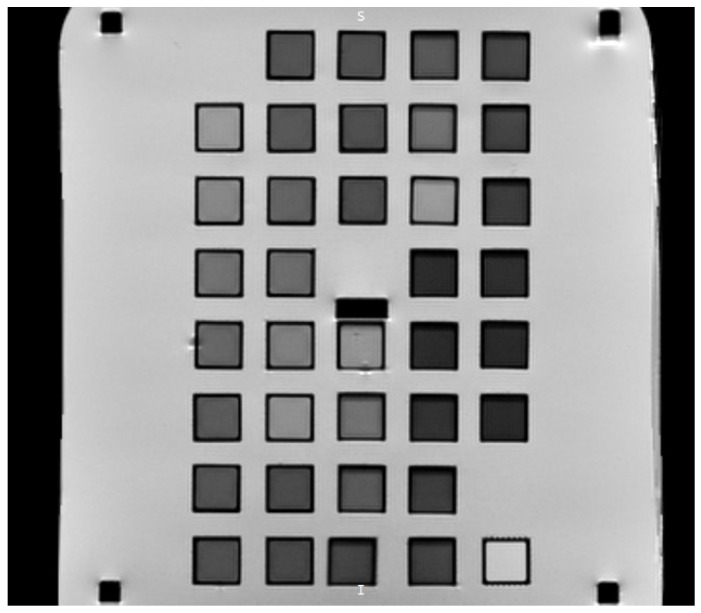
Cross-sectional MRI image of dosimetric gel samples.

The dose–response of dosimetric gel samples was analyzed 24 h after irradiation, letting radiation-induced polymerization processes to settle, because these processes continue for much longer than the irradiation time [51]. The dose response of the irradiated dosimetric gels was determined using Philips Achieva 1.5 T MRI scanner (Philips Healthcare, Best, The Netherlands). MRI was selected as the most sensitive method for acquisition of dose information, as it is not limited by high optical attenuation of the sample present at high irradiation doses [52]. T2-weighted images of the dosimetric gel samples were acquired by varying the echo time in the 20–400 ms interval. Other imaging parameters were as follows: repetition time—4000 ms, number of averages—2, slice thickness—6 mm, number of echoes—20. Head coils were used for scanning. The dose–response points (intensity values) from the MRI images were acquired by averaging the gray levels in a rectangular region representing the polymerized volume of each dosimetric gel sample (Figure 10). Since the same square-shaped region in the same position in the MRI images of dosimetric gels was analyzed, uniformity of the response of dosimetric gels was maintained despite slight changes in depth dose characteristics.

For each irradiation dose, signal intensity was plotted against echo time (TE), and T2 values (1/R2) were determined by monoexponential curve fitting. The sensitivity of dosimetric gel was considered as a slope of linear fit of dose response points.

Attenuated Total Reflectance Fourier-Transform Infrared spectroscopy (ATR-FTIR) of dosimetric gel samples was also performed. Samples irradiated with a 40 Gy dose using UHDR and CONV radiation were compared. Identical measurement conditions were maintained to avoid environmental influences on the measurements.

## Figures and Tables

**Table 1:** Uncertainty analysis.

Uncertainty Source	nPAG	nPAG + Ag	NIBMAGAT	NIBMAGAT + Ag	Uncertainty Type
Irradiation output	2%	2%	2%	2%	B
Irradiation dose evaluation	2%	2%	2%	2%	B
Sensitivity variation of imaging	3.9%	3.9%	3.9%	3.9%	A
Dose rate dependency	0.9%	2.5%	1.3%	1.8%	A
Calibration curve fit	1%	1.5%	0.4%	0.2%	A
Reproducibility	0.5%	0.4%	0.9%	0.6%	A
Combined uncertainty	5%	5.6%	5.1%	5.2%	
Total uncertainty (2σ)	10%	11.2%	10.2%	10.4%	

**Table 2:** Composition of investigated dosimetric gels.

Purpose	Material	Dosimetric Gel	
		nPAG	NIBMAGAT
Monomer	*N*-(isobutoxymethyl)acrylamide	-	2 wt%
	Acrylamide	3 wt%	-
Crosslinker	*N,N*′-methylenebis(acrylamide)	3 wt%	3 wt%
Scaffold	Gelatin	5 wt%	4 wt%
Oxygen scavenger	Tetrakis(hydroxymethyl)phosphonium chloride	5 mM	5 mM
Cosolvent	Acetone	-	30 wt%
Solvent	Water	59–89 wt%	41–71 wt%
Additive (optional)	Ag nanoparticle solution (1 mM)	30 wt%	30 wt%

## Data Availability

Material is available upon request to interested researchers.
